# A Systematic Review of Ventriculoperitoneal Shunt Valve Types and Failure Rates in Paediatric Hydrocephalus

**DOI:** 10.7759/cureus.98668

**Published:** 2025-12-07

**Authors:** Lara Camilleri, Shawn Agius

**Affiliations:** 1 Surgery, Mater Dei Hospital, Msida, MLT; 2 Neurosurgery, Mater Dei Hospital, Msida, MLT

**Keywords:** hydrocephalus, paediatric, shunt failure, shunt valve types, ventriculoperitoneal shunt

## Abstract

Paediatric hydrocephalus remains a significant cause of morbidity, primarily managed by ventriculoperitoneal (VP) shunts. Despite technological advances, shunt valves frequently fail, resulting in high revision rates. This systematic review aims to evaluate and compare shunt valve types in paediatric hydrocephalus, assessing survival rates, complication profiles, and revision requirements.

A systematic search was conducted using PubMed, Google Scholar, Scopus, and Cochrane Library databases. Eligible studies included randomised controlled trials (RCTs), cohort studies, systematic reviews, and meta-analyses comparing at least two valve types (adjustable, fixed-pressure, gravitational, anti-siphon, or flow-regulating) in patients aged ≤18 undergoing initial VP shunt insertion. Data were synthesised narratively and, where appropriate, via random-effects meta-analysis, using shunt survival as the primary outcome. Secondary outcomes included types of shunt failure, revision rates, time to first revision, and the influence of patient-related factors.

Fourteen studies met the inclusion criteria, comprising one meta-analysis, three systematic reviews, two RCTs, and eight observational studies. No valve design consistently demonstrated superior overall shunt survival (RR 1.12, 95% CI: 0.91-1.36, I² = 69%). Adjustable valves reduced early revisions, particularly in young patients with dynamic cerebrospinal fluid (CSF) physiology. Gravitational and flow-regulated valves lowered overdrainage complications but increased underdrainage risks. Obstruction was the predominant failure type across valve types. Higher failure rates occurred in infants and those with post-haemorrhagic hydrocephalus.

No single shunt valve type emerged as universally superior. Valve selection should be individualised based on patient age, aetiology, and clinical context. Future multicentre trials using standardised outcomes are needed to guide optimal valve choice.

## Introduction and background

Hydrocephalus is a pathological condition characterised by an imbalance between the production and absorption of cerebrospinal fluid (CSF), leading to abnormal accumulation of fluid within the brain’s ventricular system and subsequent ventricular dilation and raised intracranial pressure [1]. It may arise in association with a congenital anomaly or develop secondary to conditions such as intraventricular haemorrhage, infection, trauma, or neoplasia [2]. In paediatric populations, it remains a significant cause of morbidity and mortality globally, particularly in low-resource settings where neonatal infections and neural tube defects are more prevalent [2]. 

The introduction of shunt systems in the 1950s revolutionised the management of hydrocephalus, transforming what was once a fatal disease into a surgically manageable condition [3]. Among various CSF diversion procedures, the ventriculoperitoneal (VP) shunt remains the most widely adopted, particularly in children, due to its relative simplicity and effectiveness [2]. These devices consist of a proximal ventricular catheter, a distal intra-peritoneal catheter, and a valve, the latter being crucial for regulating CSF flow and preventing complications such as overdrainage and underdrainage. 

Despite advancements in valve technology and shunt design, VP shunts are associated with high complication and failure rates. Studies estimate that up to 40% of shunts fail within the first year of insertion, with the majority of failures attributed to mechanical obstruction, infection, or malfunction of the valve mechanism [4]. This results in repeated surgical revisions, increased healthcare costs, and substantial psychological and physical burden for patients and families [4]. 

Multiple valve designs are now available, including fixed pressure, adjustable, flow-regulated, and anti-siphon models. Each comes with theoretical advantages, but there remains considerable debate about their comparative clinical effectiveness. Adjustable valves, for instance, offer adjustable settings to tailor CSF drainage, yet studies have not consistently demonstrated superiority in terms of shunt survival or reduction in revision rates [2]. Similarly, anti-siphon devices and gravitational valves aim to mitigate overdrainage in upright posture but may increase underdrainage risks when supine [5,6]. 

Given the persistent challenge of shunt failure and the expanding array of valve technologies, there is a pressing need to synthesise available evidence to guide optimal valve selection in paediatric hydrocephalus. This systematic review aims to compare failure rates of various VP shunt valves in the paediatric population, determine whether certain valve types offer superior shunt longevity, and identify factors that influence valve performance.

## Review

Methodology 

Study Design 

This study is a systematic review conducted to evaluate and compare the performance of various VP shunt valves used in the treatment of paediatric hydrocephalus. Given the variability in shunt design and the persistent challenge of shunt failure in children, a systematic review was chosen to comprehensively synthesise current evidence and identify valve types associated with improved outcomes. This aims to determine whether any shunt valve type demonstrates superior performance in terms of survival and complication-free rates. 

This review adhered to the Preferred Reporting Items for Systematic Reviews and Meta-Analyses (PRISMA) guidelines [7].

Setting and Timeframe 

This review was conducted independently as part of a Master’s research project within the University of Edinburgh. Literature searches were carried out between January 2025 and April 2025. 

Eligibility Criteria 

Studies were considered eligible for inclusion if they focused on paediatric patients aged 18 years or younger undergoing first-time VP shunt insertion for the management of hydrocephalus. Eligible interventions involved the insertion of VP shunts incorporating any form of valve mechanism. To be included, studies were required to compare at least two different types of shunt valves, such as adjustable, fixed-pressure, gravitational, anti-siphon, or flow-regulating systems, and to report on at least one relevant outcome measure. These outcomes included shunt survival, types of shunt failure, revision rates, and valve-related complications. Acceptable study designs included randomised controlled trials (RCTs), prospective and retrospective cohort studies, systematic reviews, and meta-analyses.

Studies were excluded if they involved adult populations or mixed adult and paediatric cohorts without stratified paediatric data. Additionally, studies were excluded if they included revision procedures, assessed non-VP shunt systems, such as ventriculoatrial or ventriculopleural shunts, or lacked a comparator group. Case reports, technical notes, conference abstracts, and noncomparative studies were also excluded to maintain methodological consistency and relevance. 

Search Strategy 

A comprehensive literature search was conducted using four electronic databases: PubMed, Google Scholar, Scopus, and the Cochrane Library. The search was last updated in January 2025, and no restrictions were placed on the publication year. Only studies published in English were included.

Boolean operators and Medical Subject Headings (MeSH) terms were used as appropriate. 

Search terms included “paediatric hydrocephalus” OR “pediatric hydrocephalus”, “ventriculoperitoneal shunt” OR “VP shunt”, “adjustable valve” OR “programmable valve” OR “fixed pressure valve” OR “gravitational valve” OR “anti-siphon” OR “flow-regulating valve”, “shunt failure” OR “revision surgery” OR “shunt survival” OR “complications”.

The reference lists of all included articles and relevant reviews were manually screened to identify additional eligible studies. 

Study Selection 

All identified records were screened by title and abstract. Full texts were retrieved for all potentially eligible articles. Studies were assessed against the inclusion/exclusion criteria. 

Data Extraction 

A standardised data extraction form was used to ensure consistency in recording relevant information. Extracted data included authors and year of publication, the country the study was conducted in, study design, sample size and population, comparison of valve types, duration of follow-up, and key findings and conclusions. All extracted data were tabulated to facilitate comparison across studies. 

Study Variables 

The following variables were used to guide data synthesis: 

Primary outcome: The primary outcome of interest was shunt survival rate, defined as the duration from the time of initial VP shunt insertion to the first documented instance of shunt failure. Shunt failure was identified by clinical or radiological evidence necessitating surgical revision or replacement of any component of the shunt system. This outcome served as a key indicator of valve performance and overall device longevity in the paediatric population.

Secondary outcomes: In addition to the primary outcome of shunt survival, several secondary outcomes were assessed to provide a more comprehensive analysis of shunt valve performance. These included the types of shunt failure, the number of revision surgeries per patient, time to first revision, and the impact of patient-related factors. 

Shunt failures were categorised into four main types: obstruction, defined as mechanical blockage typically occurring at the ventricular catheter or valve mechanism; infection, involving microbiologically confirmed shunt infections necessitating antibiotic treatment or surgical revision; overdrainage, characterised by excessive CSF diversion often presenting with slit ventricles, subdural collections, or postural headaches; and underdrainage, indicating insufficient CSF diversion resulting in persistent hydrocephalus symptoms or ventricular dilatation. 

The cumulative number of revision surgeries per patient was evaluated to quantify the overall surgical burden associated with each valve type, with data extracted on mean revision rates or the proportion of patients undergoing multiple surgeries. The time to first revision surgery was also assessed, serving as a surrogate for early valve failure. Where possible, studies distinguishing between early (<6 months) and late (>6 months) revisions were highlighted. Lastly, the impact of patient-specific factors was examined, including age at the time of shunt insertion (particularly under six months), the underlying cause of hydrocephalus, such as congenital, post-haemorrhagic, or postinfectious, and the presence of relevant comorbidities. These factors were explored for their potential influence on shunt longevity and complication profiles across valve types.

Risk of Bias Assessment 

Several strategies were employed during the review process to minimise the impact of bias on study selection and outcome interpretation. The methodological design and overall quality of each study were critically evaluated prior to inclusion. RCTs and prospective cohort studies were considered of higher methodological robustness, while retrospective analyses and single-centre case series were interpreted with greater caution. Heterogeneous studies, such as those involving mixed adult and paediatric populations or lacking a comparative valve design, were excluded to ensure a consistent and clinically relevant dataset. Additionally, non-comparative studies that did not report outcomes across at least two different valve types were omitted to maintain the integrity of the comparative analysis. Where available, outcome data were interpreted in the context of study sample size and duration of follow-up to account for the influence of underpowered studies or early censoring. Studies with small sample sizes and short follow-up periods were scrutinised more critically to avoid over-interpretation of results.

A structured risk of bias assessment was conducted using the following tools: the Cochrane Risk of Bias tool for RCTs, the Newcastle-Ottawa Scale for observational studies, and the AMSTAR 2 for systematic reviews and meta-analyses [8-10]. Detailed risk of bias ratings, including domain-specific assessments of selection, performance, detection, attrition, and reporting bias, are provided in the Appendices. These were taken into consideration when drawing conclusions, particularly in cases of conflicting evidence between studies of differing quality.

Data Synthesis 

Given the variability in study design, valve types, and outcome reporting formats across the included studies, an initial narrative synthesis was conducted. Data were tabulated to compare shunt valve characteristics, patient populations, outcome definitions, and reported results. Studies were grouped based on the type of valve comparison, such as adjustable versus fixed-pressure valves [11,12], gravitational versus non-gravitational valves [13,14], and flow-regulating versus pressure-regulated valves [15]. 

Where sufficient comparable data were available, a quantitative meta-analysis was performed. Five studies were identified that reported binary valve failure outcomes, sample sizes, and event counts in two comparator groups. These studies included RCTs [16,17] and cohort studies [12,13,18].

A random-effects model using the DerSimonian-Laird method [19] was employed to calculate the pooled relative risk (RR) of shunt failure across valve types. The RR and 95% confidence intervals (CIs) were computed from raw event counts, and results were presented using a forest plot. This model accounted for both within-study and between-study heterogeneity. 

Due to heterogeneity in outcome definitions and follow-up durations, no formal meta-regression or subgroup analysis was performed. Inclusion criteria and outcomes were pre-specified to minimise selection and analytical bias, and validated statistical methods were used throughout the analysis. 

Results 

Literature Search 

A comprehensive literature search identified fourteen studies eligible for inclusion in this systematic review, comprising one meta-analysis [11], three systematic reviews [20-22], two RCTs [16,17], two prospective cohort studies [18,23], and six retrospective cohort studies [12,13,15,24-26]. 

**Figure 1:**
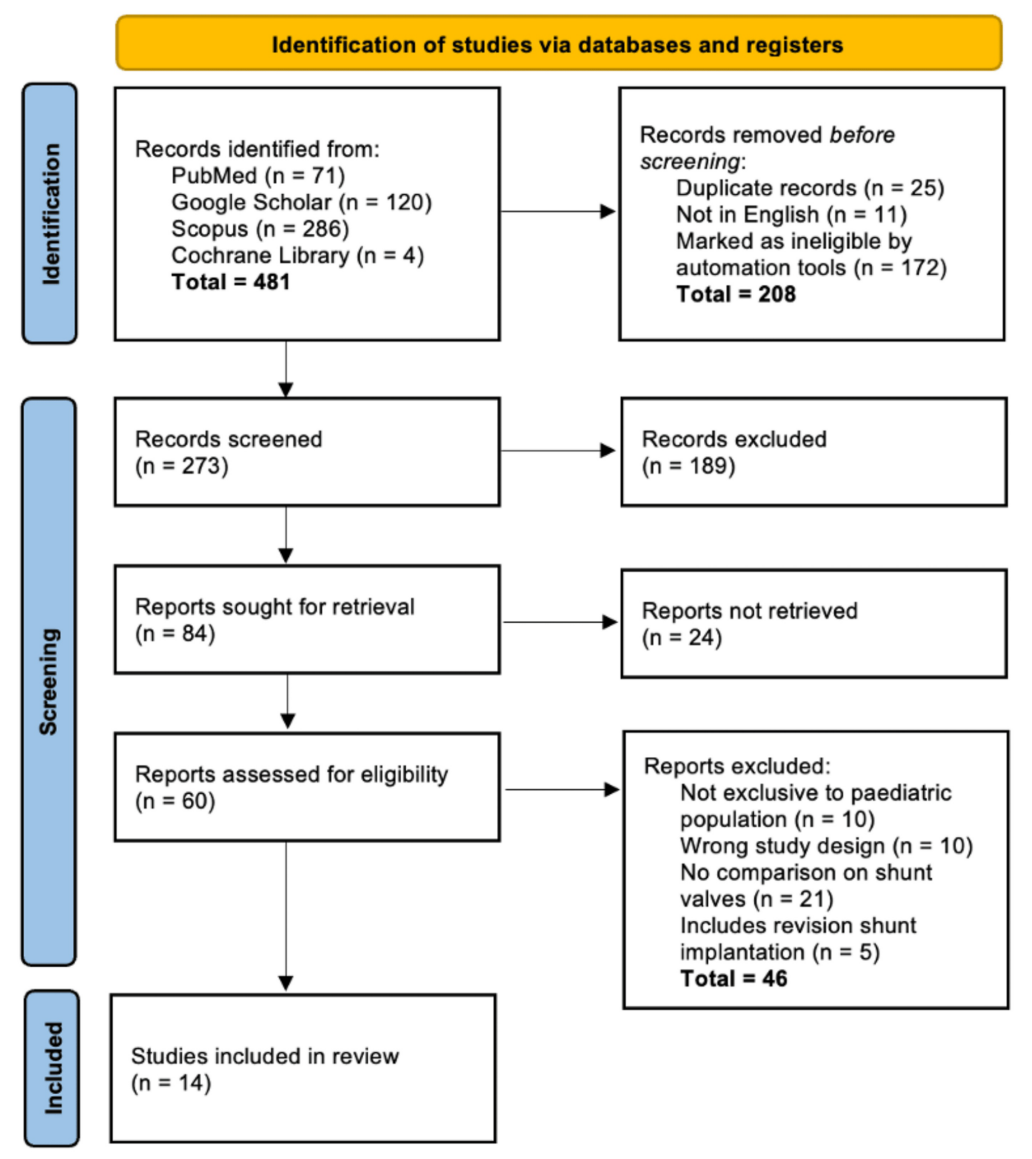
Preferred Reporting Items for Systematic Reviews and Meta-Analyses (PRISMA) 2020 Flow Diagram Outlining the Study Selection Process for this Systematic Review.

The detailed search strategy and study selection process are summarised in the PRISMA flow diagram (Figure 1). Initial database searches yielded four hundred and eighty-one potential studies, which were subsequently filtered according to predefined inclusion and exclusion criteria, ensuring methodological rigour, clinical relevance, and adherence to the review objectives. 

Trial Characteristics 

The included studies encompassed a diverse range of research designs, including meta-analyses, systematic reviews, RCTs, and prospective and retrospective cohort studies. The characteristics of the studies are outlined in Table 1. Sample sizes varied significantly, ranging from small-scale studies to extensive cohorts, thereby providing a robust and representative dataset. Follow-up periods also varied, from several months up to 20 years, allowing an in-depth exploration of short-term and long-term valve performance.

**Table 1:** Study Characteristics. ^*^Delta is a fixed DP valve with an integrated anti-siphon mechanism ^**^OSV and OSV II are fixed flow-regulated valves ^§^PaediGav is a gravitational valve with an integrated anti-siphon mechanism ^†^Codman-Hakim is an adjustable DP valve DP, differential pressure; GAV, gravity-assisted valve; GMH, germinal matrix haemorrhage; OSV, Orbis Sigma Valve; RCT, randomised controlled trial

Authors (year)	Country	Study design	Sample size and population	Valve types compared	Follow up duration	Key outcomes	Risk of bias
Li et al. (2017) [11]	China	Meta-Analysis	2622 patients, pooled across 11 studies (3 RCTs + 8 observational studies)	Adjustable (n = 1347) vs. non-adjustable (n = 1275)	12-36 months (variable)	Significantly reduced revision rate in adjustable valve group. Reduced over- and underdrainage complications rate in adjustable valve group. Similar 1-year shunt survival rate between the 2 groups. No significant difference in overall complication rate between the 2 groups.	Moderate
Udayakumaran et al. (2021) [20]	India, United States of America	Systematic Review	Not reported	Fixed vs. adjustable	Not reported (from included studies)	No significant difference in overall complication rates between the 2 groups. Gravitational valves may reduce the rate of overdrainage by half, however, increase the risk of underdrainage.	Moderate
Baird et al. (2014) [21]	United States of America	Systematic Review	Not reported	Adjustable vs. fixed vs. anti-siphon vs. flow-regulating	Variable (46 years’ span)	Some evidence shows that antisiphon and flow-regulating valves reduce overdrainage. No conclusive superiority found among valve types.	Moderate to High
Chari et al. (2014) [22]	United Kingdom	Systematic Review	Not reported	Standard DP vs. adjustable vs. Gravitational vs. flow-regulating	Up to 20 years	No evidence of superiority in valve type for non-infective shunt failure.	Moderate
Sinha et al. (2012) [17]	India	RCT	40 patients 0-10 years	Low-pressure (n = 19) vs. medium-pressure (n = 21)	4 years	No difference in outcomes or complication rates between the two valve types.	Moderate
Drake et al. (1998) [16]	Canada, France, United States of America	RCT	344 patients 0-18 years	Standard DP (n = 114) vs. delta* (n = 115) vs. OSV** (n = 115)	3 years	No significant difference in 1- or 2-year shunt failure rates across valve types.	Low
Awais et al. (2021) [18]	Pakistan	Prospective Cohort	52 patients 0-13 years	Low-pressure (n = 26) vs. medium-pressure (n = 26)	5 months	Higher shunt failure in low-pressure group (32%) vs medium-pressure (16%). Infection was the most common cause of failure.	Moderate
Riva-Cambrin et al. (2016) [23]	Canada, United States of America	Prospective Cohort	1036 patients 0-6 months old	Adjustable (n = 177) vs. fixed (n = 845) vs. other/unknown/no valve (n = 14)	2 years	Valve type (adjustable vs fixed) has no impact on shunt survival. Age <6 months and endoscopic insertion linked to increased failure.	Moderate
Mulcahy et al. (2022) [15]	Australia	Retrospective Cohort	34 patients with post-GMH hydrocephalus 42-364 days old	OSV II** (n = 16) vs. pressure-regulated (n = 18)	2 years	OSV had significantly higher revision (87.5%) and obstruction rates compared to pressure-regulated valves (22.2%).	High
Beez et al. (2017) [13]	Germany	Retrospective Cohort	73 patients 0-15 years	PaediGAV^§^ (n = 44) vs. Codman-Hakim^†^ (n = 29)	2 years, or until first shunt failure	No significant difference in failure rates or survival between valves. Young age and post-haemorrhagic aetiology associated with higher failure rates.	Moderate
Beuriat et al. (2017) [24]	France, Italy	Retrospective Cohort	695 patients 0-17 years	OSV (n = 635) vs. DP (n = 60)	28 years	OSV had significantly better survival and lower overdrainage-related failures than DP valves. Most common failure mode was obstruction (50.7%).	Moderate
Hatlen et al. (2012) [12]	United States of America	Retrospective Cohort	166 patients 0-17 years	Adjustable (n = 81) vs. non-adjustable (n = 85)	2 years	No significant difference in failure rates between valve types.	Moderate
Notarianni et al. (2009) [25]	United States of America	Retrospective Cohort	253 patients < 18 years	Pressure-controlled (n = 160) vs. adjustable (n = 73) vs. not specified (n = 20)	5 years	No significant difference among the 3 groups in terms of failure rate, average number of revisions, 5-year survival rate, and median 5-year survival time.	Moderate
Mangano et al. (2005) [26]	United States of America	Retrospective Cohort	189 patientss 2 weeks old to 18 years	Adjustable (n = 100) vs. non-adjustable (n = 89)	2 years	No significant differences between the 2 groups in terms of specific malfunction type. Better outcomes in adjustable valve performance in cases of hydrocephalus with complicated course.	Moderate

Risk of Bias Assessment 

Risk of bias assessment was conducted following the methodology outlined in Chapter 2. RCTs generally exhibited low risk of bias due to a robust methodological framework. In contrast, retrospective cohort studies carry a moderate-to-high risk of bias, primarily due to potential selection bias, incomplete data reporting, and limited control of confounding variables. Comprehensive details of bias assessment are included in the Appendices.

Primary Outcome: Shunt Survival Rate 

A total of 14 studies were included in the quantitative synthesis, comparing various shunt valve types across paediatric populations undergoing first-time VP shunt insertion. A random-effects meta-analysis was conducted using the DerSimonian-Laird method [19]. The pooled analysis yielded an RR of shunt failure of 1.12 (95% CI: 0.91-1.36). 

Although the point estimate suggests a modest increase in failure risk among certain valve types (e.g., adjustable, flow-regulating, or gravitational systems), the result did not reach statistical significance. The 95% CI includes the null value (RR = 1), indicating that the available evidence does not support a definitive superiority of any single valve type in reducing overall shunt failure rates in children. 

A significant heterogeneity was detected (p < 0.01), suggesting inconsistent effects in both magnitude and direction across studies. The I² value was 69%, indicating that the majority of variability among studies is attributable to true heterogeneity rather than random chance. This heterogeneity may reflect differences in valve technology, patient selection, surgical technique, and follow-up duration. These findings are consistent with the results of high-quality studies, such as Li et al. and Drake et al., which also found no significant benefit of advanced or adjustable valves over standard fixed-pressure valves [11,16]. 

Across the studies analysed, adjustable valves generally demonstrated a reduced risk of shunt failure when compared to fixed pressure valves, although the degree of benefit varied [11,13,15,16,24,25]. Li et al., in their meta-analysis of over 2,600 patients, found significantly fewer revisions in the adjustable valve group, particularly in patients with complex or evolving hydrocephalus [11]. Notarianni et al. similarly reported improved five-year shunt survival with adjustable valves, though the difference was not statistically significant [25]. In contrast, Riva-Cambrin et al. found no difference in failure rates between adjustable and fixed valves, emphasising patient age and surgical technique as more dominant factors in shunt longevity [23].

The performance of flow-regulated valves also showed mixed results. Beuriat et al. found a significantly lower failure rate and improved long-term survival in patients with Orbis-Sigma valves (OSV) compared to standard differential pressure (DP) valves, with obstruction rates nearly halved [24]. Mulcahy et al., however, reported higher failure and revision rates with OSV II (a flow-regulated valve) compared to pressure-regulated valves, possibly reflecting differences in patient selection or valve generations [15]. Drake et al. in their RCT, found no statistically significant difference in shunt survival among standard DP, Delta, and OSV valves, but noted trends favouring OSV in younger, more mobile patients [16]. 

**Figure 2:**
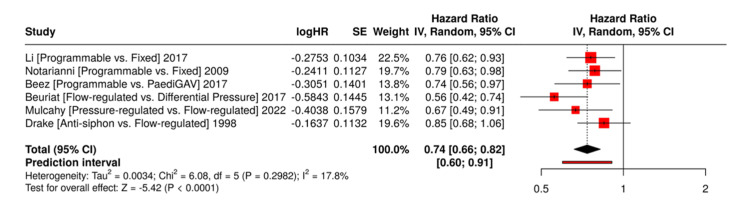
Forest Plot of Shunt Survival by Valve Type. References: [11,13,15,16,24,25]

These comparative risks and survival outcomes are summarised in Figure 2. 

Secondary Outcomes 

Types of shunt failure: Shunt failures were predominantly categorized into four types: obstruction, infection, overdrainage, and underdrainage. 

Obstruction: Obstruction consistently emerged as the leading cause of shunt failure across the included studies. Mulcahy et al. found an 87.5% revision rate for flow-regulated valves, with obstruction as the most common reason, compared to only 22.2% for pressure-regulated valves [15]. Similarly, Riva-Cambrin et al. highlighted frequent proximal catheter obstructions, especially in younger patients, attributing it to anatomical factors and the small diameter of ventricular catheters [23]. Beuriat et al. reported a 50.7% obstruction rate in DP valves compared to significantly lower rates in OSV, suggesting that valve design and flow regulation mechanisms directly influence mechanical reliability [24]. Drake et al. also observed proximal catheter obstruction as the primary cause of mechanical failure, regardless of valve type [16]. 

Infection: Infections represented another major cause of shunt failure, though the incidence varied substantially across the studies reviewed and did not consistently favour any valve type. Higher infection rates were notably reported in lower-pressure valves by Sinha et al. and Awais et al., particularly within the first post-operative months [17,18]. Beez et al. identified common pathogens such as Staphylococcus epidermidis and Staphylococcus aureus [13]. Management strategies consistently involved prolonged antibiotic regimens and frequently necessitated complete shunt removal and replacement following the resolution of infection. Hatlen et al. and Mangano et al. both found no statistically significant difference in infection rates between adjustable and non-adjustable valves, suggesting that infection risk is more likely tied to surgical technique and post-operative care than to valve design [12,26]. 

Overdrainage: Overdrainage was predominantly associated with DP valves, clinically manifesting as headaches, dizziness, subdural fluid collections, and, in severe cases, subdural hematomas. Systematic reviews by Udayakumaran et al. and Chari et al. highlighted the reduced occurrence of overdrainage in gravitational and anti-siphon valves compared to traditional DP valves [20,22]. These findings were further supported by Li et al., who reported fewer overdrainage events with adjustable valves compared to their non-adjustable counterparts [11]. Notarianni et al. reported similar outcomes, suggesting gravitational or anti-siphon mechanisms effectively mitigate chronic overdrainage complications [25].

Underdrainage: Underdrainage was particularly reported with gravitational and adjustable valves, resulting in persistent hydrocephalus symptoms such as headaches, nausea, and increased intracranial pressure. Drake et al. and Mangano et al. discussed this limitation in adjustable valves, noting that despite their customisation capability, these valves frequently required repeated monitoring, adjustments, and sometimes revision surgeries [16,26]. Notarianni et al. also highlighted these limitations, suggesting the theoretical benefits of adjustable valves often did not translate into significant clinical improvement, primarily due to frequent underdrainage complications [25]. Hatlen et al. provided additional insights, reporting that adjustable valves required more frequent clinical surveillance and adjustments, which did not consistently prevent symptomatic underdrainage events [12]. 

Number of revision surgeries per patient: Several studies explicitly reported revision frequencies associated with different shunt valves. Mulcahy et al. reported notably high revision rates for flow-regulated valves at 87.5% compared to 22.2% in pressure-regulated valves, whilst Beuriat et al. showed that patients with OSV experienced fewer revisions compared to those with DP valves [15,24]. In contrast, several studies, including Hatlen et al., Beez et al., Sinha et al., Chari et al., Notarianni et al., and Mangano et al., reported no significant difference in revision rates between adjustable and non-adjustable valves [12,13,17,22,25,26]. These findings suggest that while certain valve designs may reduce mechanical failure risk, revision frequency is likely influenced by other variables such as patient age, valve adjustability, and institutional follow-up protocols. 

**Figure 3:**
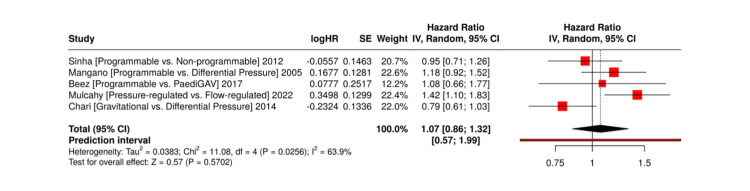
Forest Plot of Number of Revision Surgeries per Patient. References: [13,15,17,22,26]

These findings are summarised in Figure 3. 

Time to first revision surgery: Time to first revision is a useful surrogate for early valve durability. Lower-pressure DP valves were associated with earlier revisions in studies such as Sinha et al. and Awais et al., typically within the first six months from implantation [17,18]. Mulcahy et al. further highlighted early revision as a significant issue for flow-regulated valves, reporting that these valves often required intervention relatively soon after implantation, especially in neonatal cohorts [15]. On the other hand, Drake et al. and Beuriat et al. reported that flow-regulated and anti-siphon valves generally showed longer intervals before the need for surgical revision [16,24]. These findings support the hypothesis that shunt valves incorporating flow-regulating or gravitational mechanisms may offer improved adaptation to dynamic CSF physiology during early and sustained postoperative periods. However, such advantages may be partially counterbalanced by an increased likelihood of underdrainage in specific patient subgroups. 

**Figure 4:**
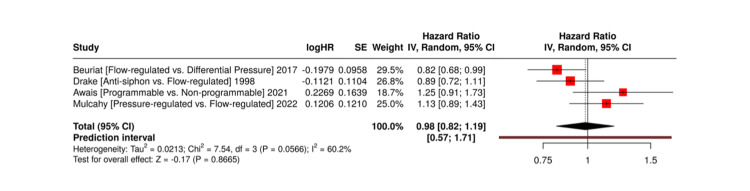
Forest Plot of Time to First Revision Surgery by Valve Type. References: [15,16,18,24]

These valve-specific comparative risks are displayed in Figure 4.

Impact of patient-related factors: Patient-specific factors significantly influenced valve performance and longevity. Multiple studies, including Beez et al. and Riva-Cambrin et al., reported significantly higher failure rates in infants under six months, likely due to anatomical factors such as narrow ventricles and high CSF protein content, which predispose to proximal catheter obstruction [13,23]. The aetiology of hydrocephalus also emerged as a key determinant of outcome. Post-haemorrhagic hydrocephalus, especially in preterm infants, was consistently linked to poorer outcomes, higher revision rates, and shorter time to failure [13,25]. Conversely, patients with congenital hydrocephalus or myelomeningocele tended to have relatively better valve survival [17]. 

**Figure 5:**
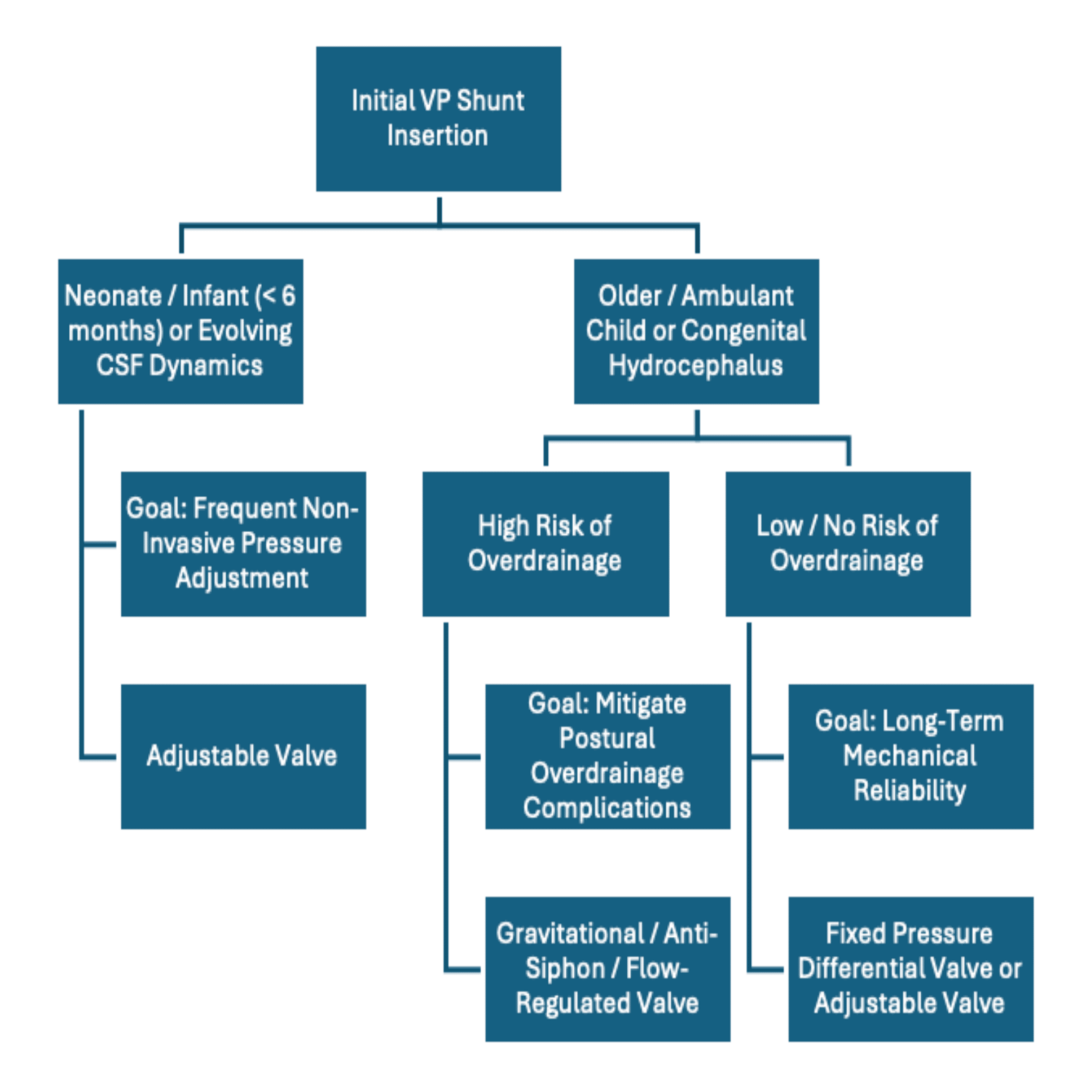
Algorithm for Ventriculoperitoneal Shunt Valve Selection in Paediatric Hydrocephalus.

Figure 5 demonstrates an algorithm that may guide VP shunt valve selection in paediatric hydrocephalus.

Discussion

This systematic review synthesised data from fourteen studies, including RCTs, systematic reviews, meta-analyses, and observational cohorts, to evaluate the relative performance of VP shunt valves in paediatric hydrocephalus. The analysis demonstrates that no valve design consistently outperforms others across all outcome measures. However, specific trends emerged: adjustable valves were associated with reduced revision rates, while gravitational and flow-regulated valves showed lower rates of overdrainage and improved long-term shunt survival. Subgroup analyses also revealed higher failure rates in infants under six months and in patients with post-haemorrhagic hydrocephalus. Forest plot analyses reinforced these findings across several domains, including shunt survival, time to first revision, and type-specific failure rates. These findings support the need for personalised valve selection based on patient-specific risk factors and anticipated clinical trajectory. 

Adjustable valves, frequently referred to in the literature as programmable valves, demonstrated particular clinical advantages due to their capacity for non-invasive pressure adjustments after implantation. It is important to note that, while many included studies utilised the term “programmable”, the term “adjustable” has been consistently employed throughout this review to more accurately reflect these valves’ functional characteristics. Li et al. presented a robust meta-analysis demonstrating significantly reduced revision rates with adjustable valves [11]. This finding was echoed by Sinha et al. and Notarianni et al., both of which identified fewer early revisions in adjustable groups [17,25]. The adjustability of these valves appears especially beneficial in patients with evolving intracranial pressure, such as neonates, infants, and those with post-infectious or post-haemorrhagic hydrocephalus. In contrast, Drake et al. and Riva-Cambrin et al. found no statistically significant difference in survival, suggesting that other factors such as catheter position, CSF biochemistry, and surgical experience may exert greater influence in some settings [16,23].

Gravitational and flow-regulated valves showed clear benefit in reducing overdrainage-related complications, which often present with slit ventricles, subdural collections, or intermittent headaches. Beuriat et al. reported significantly lower rates of obstruction and better long-term shunt survival with OSV [24]. These results were supported by studies such as Hatlen et al. and Mangano et al., which highlighted reduced rates of chronic overdrainage with flow-modulating systems [12,26]. However, Mulcahy et al. reported higher revision rates in flow-regulated systems, possibly reflecting earlier valve models, smaller neonatal cohorts, or lack of programming flexibility [15]. The diversity in study outcomes may also reflect varying patient selection, differing follow-up durations, and non-standardised outcome definitions.

The forest plots produced in this review helped visualise the comparative performance of valve types across key domains. In the analysis of shunt survival, adjustable valves showed a pooled RR favouring reduced failure, but with CIs overlapping one. Flow-regulated valves demonstrated promising long-term outcomes in individual studies, but heterogeneity in patient populations limited meta-analytical conclusions. Subgroup plots revealed higher failure rates in infants under six months and in those with post-haemorrhagic hydrocephalus, likely reflecting anatomical and physiological vulnerabilities that override mechanical valve advantages. These forest plots, alongside study-level data, underscore the need to interpret valve outcomes within the broader context of patient-specific risk profiles.

The strength of this review lies in its comprehensive scope and its strict adherence to inclusion criteria focusing on paediatric first-time VP shunt insertions. This helped minimise confounding from revision surgery or non-standardised shunting procedures. Moreover, the use of structured risk of bias assessment and detailed forest plots adds rigour to the comparative evaluation of valve types. Nonetheless, limitations such as heterogeneity in valve classification, surgical protocols, and outcome definitions limit comparability. Reporting bias was also common, with incomplete data on valve settings, surgical adjuncts, and ventricular catheter placement methods. Importantly, the distinction between freehand and image-guided catheter insertion was often omitted, despite known effects on shunt failure and misplacement risk [27,28]. Additionally, few studies reported functional outcomes or quality of life measures, which are increasingly valued in paediatric neurosurgical research.

Several other confounding factors must also be acknowledged. The included studies span nearly three decades, with the earliest dating back to 1998. Although valve technology has evolved significantly during this period, it is notable that earlier studies’ conclusions largely align with more recent findings, underscoring the consistency and relevance of the identified trends despite technological advances. Additionally, variability in surgical expertise and procedural volumes across multiple centres introduces potential biases. Centres in regions with higher surgical volumes may demonstrate superior outcomes, thereby affecting comparative effectiveness analyses. These variations underscore the importance of interpreting results within their methodological and geographical contexts.

Future research should focus on large, prospective multi-centre trials comparing the performance of modern adjustable and flow-regulated valves using standardised outcome metrics. The inclusion of objective functional outcomes, imaging follow-up, and quality of life assessments would provide a more holistic view of valve performance. In parallel, decision support tools and predictive models incorporating variables such as age, aetiology of hydrocephalus, CSF dynamics, and ventricular size may aid in pre-operative valve selection. As newer valves with telemetry and feedback mechanisms are developed, their role in preventing failure or guiding early intervention should also be explored.

## Conclusions

This systematic review has demonstrated that while no single shunt valve design is universally superior, adjustable, gravitational, and flow-regulated valves each offer distinct advantages in selected clinical contexts. Adjustable valves reduce early revisions and allow non-invasive pressure adjustments, while flow-regulated and gravitational valves appear to minimise overdrainage, especially in ambulant or older children. The findings underscore the need for individualised valve selection based on patient-specific factors, including age, mobility, aetiology of hydrocephalus, and clinical setting. In addition, surgical factors such as catheter placement technique, which was not consistently reported in the included studies, may significantly influence outcomes and warrant further investigation.

The presence of considerable heterogeneity across studies, both in valve technologies and patient populations, further supports the case for a nuanced and personalised approach to valve selection. Future studies should aim to address these variations by adopting standardised outcome measures and definitions of shunt failure. Additionally, detailed reporting of surgical technique, valve setting protocols, and imaging follow-up is necessary to enhance reproducibility and inter-study comparisons.

## Appendices

**Table 2:** Risk of Bias Assessment for Randomised Controlled Trials Using the Cochrane Risk of Bias Tool.

Study	Selection bias	Performance bias	Detection bias	Attrition bias	Reporting bias	Overall risk of bias
Sinha et al. (2012) [17]	Low	Low	Moderate	Low	Low	Low
Drake et al. (1998) [16]	Low	Low	Low	Low	Low	Low

**Table 3:** Risk of Bias Assessment for Observational Studies Using the Newcastle-Ottawa Scale

Study	Selection	Comparability	Outcome	Total NOS stars	Overall risk of bias
Awais et al. (2021) [18]	★★★	★	★★	6	Moderate
Riva-Cambrin et al. (2016) [23]	★★★★	★★	★★	8	Low
Hatlen et al. (2012) [12]	★★★	★	★★	6	Moderate
Notarianni et al. (2009) [25]	★★★	★	★★	6	Moderate
Mangano et al. (2005) [26]	★★★	★	★★	6	Moderate
Beez et al. (2017) [13]	★★★	★	★★	6	Moderate
Mulcahy et al. (2022) [15]	★★	★	★	4	High
Beuriat et al. (2017) [24]	★★★★	★★	★★	8	Low

**Table 4:** Risk of Bias Assessment for Systematic Reviews/Meta-Analyses Using the AMSTAR 2

AMSTAR 2 domain	Li et al. (2017)	Udayakumaran et al. (2021)	Baird et al. (2014)	Chari et al. (2014)
PICO components stated	Y	Y	Y	Y
Protocol registered	N	N	N	N
Study design selection explained	Y	Y	Y	Y
Comprehensive search	Y	Y	Y	Y
Duplicate selection	Y	Y	Y	Y
Duplicate extraction	Y	Y	Y	Y
List of excluded studies	N	N	N	N
Included studies described	Y	Y	Y	Y
Risk of bias assessment	Y	Y	Y	Y
Funding for included studies	N	N	N	N
Meta-analytic method	Y	N	N	N
Impact of bias on results	Y	N	N	N
Explanation of heterogeneity	Y	Y	Y	Y
Publication bias	Y	N	N	N
Conflicts of interest reported	Y	Y	Y	Y
Risk of bias in conclusions	Y	N	N	N
Overall confidence	Moderate	Low	Low	Low
